# Surgeons’ knowledge regarding the diagnosis and management of pancreatic cancer in China: a cross-sectional study

**DOI:** 10.1186/s12913-017-2345-6

**Published:** 2017-06-09

**Authors:** Bing-Qi Li, Li Wang, Jian Li, Li Zhou, Tai-Ping Zhang, Jun-Chao Guo, Yu-Pei Zhao

**Affiliations:** 1 Department of General Surgery, Peking Union Medical College Hospital, Chinese Academy of Medical Sciences/Peking Union Medical College, Beijing, China; 2 Department of Epidemiology, Institute of Basic Medicine, Peking Union Medical College & Chinese Academy of Medical Science, Beijing, China

**Keywords:** Surgeons’ knowledge, Pancreatic cancer, Cross-sectional study

## Abstract

**Background:**

Pancreatic cancer is rare but highly malignant. Studies have shown that surgeons’ knowledge closely links to the correct diagnosis and treatment outcomes of pancreatic cancer. The purpose of this study was to survey current surgeons’ knowledge regarding pancreatic cancer.

**Methods:**

A cross-sectional study was conducted among 705 surgeons who attended the 2011 China Surgical Week’s meeting in Beijing. A questionnaire regarding the risk factors, clinical manifestations, diagnosis, and treatment of pancreatic cancer was used. Surgeons’ answers were analyzed and compared among different regions, levels of hospital, and professional ranks.

**Results:**

Most surgeons had a correct knowledge toward the risk factors, diagnosis, and management of pancreatic cancer. However, several knowledge gaps were identified. They include “The association between type 2 diabetes and pancreatic cancer”, “The most common histologic type of pancreatic neoplasm”**,** “the typical clinical symptoms of pancreatic cancer”, “The accuracy of ultrasound in screening pancreatic cancer”, “Enhanced CT in the diagnosis of pancreatic cancer”, and “Which is more superior between MRI and CT in the diagnosis of pancreatic cancer”. We also found that overall surgeons’ responses did not depend on their geographic locations, but on hospital levels and professional ranks. Surgeons working at level three hospitals had better knowledge than others in certain areas and resident surgeons had fewer correct answers in some areas.

**Conclusions:**

Although most surgeons have a good knowledge in most areas related to the diagnosis and treatment of pancreatic cancer in China, certain knowledge gaps exist, particularly among trainees and those from low level hospitals. Continuing medical education programs to improve these knowledge gaps should be implemented.

**Electronic supplementary material:**

The online version of this article (doi:10.1186/s12913-017-2345-6) contains supplementary material, which is available to authorized users.

## Background

Pancreatic cancer (PC) is the eighth most common cause of cancer death worldwide, while its incidence ranks thirteenth. The mortality to incidence ratio is nearly 0.98:1 [1]. Based on reports from China’s Disease Surveillance Point System, the age-adjusted mortality rate has increased from 2.18 per 100,000 population in 1991 to 3.26 per 100,000 population in 2000 [2]. As the Chinese population becomes increasingly urbanized and aged, it is expected that the incidence of PC in China will continue to increase over the next several decades. Due to its highly aggressive biological behaviour and difficulties in early diagnosis, PC is still a disease with a very poor prognosis. Its 5-year survival is less than 5% [3]. Comprehensive treatment consisiting of radical surgical resection remains the mainstream treatment for PC today.

Comprehensive treatment for cancer requires not only technical skills, but also the knowledge of oncology relative to PC. In China, surgeons are responsible for making decisions regarding the preoperative, intraoperative, and postoperative management of PC. Therefore, they play a central role in the improvement of survival of PC patients. Studies have shown that physician’s knowledge contributes to the quality of care [4, 5]. Incorrect knowledge of surgeons may bring barriers to the improvement of PC patients’ survival. “The guidelines for the diagnosis and treatment of pancreatic cancer in China” has been available since 2007 [6]. But surgeons’ knowledge and opinions towards the diagnosis and treatment of PC in China remain variable. Understanding the gap between surgeons’ knowledge and the guidelines may help improve care and survival of these patients. With the goal of standardizing and improving the treatment of PC, we therefore performed such a survey using questionnaire which can help us acquire general situation about surgeons’ mastery of the guideline of PC.

## Methods

The ethical committee of Peking Union Medical College Hospital granted approval to conduct this study. Written informed consents have been obtained from the participants. Participation in the survey was voluntary. The privacy of all participants was protected at all times. Questionnaires were stored in a locked filing cabinet and the data were stored on a password protected computer.

A questionnaire [see Additional file 1] assessing surgeons’ knowledge about the diagnosis and treatment of PC was distributed at the registration desk at the 2011 China Surgical Week’s meeting in Beijing. There were 8350 physicians and surgeons from hepato-biliary-pancreatic, gastrointestinal or general surgery departments registered for the meeting. After randomized sampling from the attendee’s list, 850 surgeons were enrolled in the study.

All the items in the questionnaire were created according to the guidelines for the diagnosis and treatment of pancreatic cancer [6]. The survey contained five sections. The first section was demographics of the participants, including professional rank, specialty, practice hospital and hospital’s level. The second section included the general information as well as the risk factors of PC. The third section consisted of questions regarding clinical manifestations. The forth section contained questions regarding the diagnosis, including staging, tumor markers, imaging modalities, and other information related to the assessment of resectability of PC. The fifth section addressed issues related to the management of PC, including preoperative procedures, operative details, and postoperative management. Most of the sections used “agree”, “disagree” and “uncertain” to assess the surgeons’ knowledge. A total of 850 Questionnaires were offered and 725 were returned. After excluding 20 questionnaires that missed more than 20% of the questions, 705 questionnaires were included for the analysis.

The participants were grouped into 3 different regions based on their geographic locations (i.e., eastern, central and western regions) and economic development level. The eastern region has the highest level and the western has the lowest. Correct answers were compared among surgeons with different professional ranks, at different levels of hospitals and in different regions. Pearson chi-square or fisher exact test was used to compare categorical variables when appropriate. If there was difference observed among the different groups, two by two comparison was used to determine the exact groups after false discovery rate (FDR) adjustment. A statistical software SAS9.2 (SAS institute, Cary, NC, USA) was used for the statistical analysis.

## Results

### Participant demographics

Table 1 summarized the demographic characteristics of 705 surgeons who were included in the survey. Most surgeons worked at level 3 hospitals (85.5%) and the rest worked at level 2. Surgeons with the rank of chief physician, associate chief, attending physician, and resident accounted for 31.8, 31.5, 17.9 and 18.9%, respectively. Over half (58.2%) of the surgeons were from hospitals in the Eastern region, 24.0% were from central, and 17.9% were from Western region. The proportions of surgeons who specialized in pancreatic surgery, hepato-biliary surgery, gastrointestinal surgery, and general surgery were 28.8, 35.0, 16.7 and 13.6%, respectively. The distribution of practice hospitals, professional ranks, and subspecialties among the surgeons from eastern, central and western China were not significantly different. There was no signifianct difference of demographic characteristic between the 705 and 20 physicians not included in the data analysis [see Table 2].

**Table 1:** Characteristics of participants in the study

	*Total*	Eastern area	Central area	Western area	*P*
*Total*	705	410(58.2)	169(24.0)	126(17.9)	
Practice hospital(%)					
Level 2	102(14.5)	69(16.8)	18(10.7)	15(11.9)	*0.105*
Level 3	603(85.5)	341(83.2)	151(89.3)	111(88.1)	
Major(%)					
Pancreatic surgery	210(29.8)	122(29.8)	48(28.4)	40(31.8)	0.886
Hepato-biliary surgery	247(35.0)	143(34.9)	61(36.1)	43(34.1)	
Gastrointestinal surgery	118(16.7)	72(17.6)	26(15.4)	20(15.9)	
General surgery	96(13.6)	50(12.2)	27(16.0)	19(15.1)	
Missing	34(4.8)	23(5.6)	7(4.1)	3(3.2)	
Professional titles(%)					
Resident	133(18.9)	74(18.0)	43(25.4)	16(12.7)	0.067
Attending physician	126(17.9)	70(17.1)	26(15.4)	30(23.8)	
Associate chief physician	222(31.5)	127(31.0)	51(30.2)	44(34.9)	
Chief physician	224(31.8)	139(33.9)	49(29.0)	36(28.6)

**Table 2:** The demopraphic chacteristic comparison between the physicians included and not included in the data analysis

	Included	Not included	P
Total	705	20	
Practice hospital (%)			
Level 2	102(14.5)	3(15.0)	1.000
Level 3	603(85.5)	17(85.0)	
Major (%)			
Pancreatic surgery	210(29.8)	10(50.0)	0.233
Hepato-biliary surgery	247(35.0)	3(15.0)	
Gastrointestinal surgery	118(16.7)	3(15.0)	
General surgery	96(13.6)	3(15.0)	
Missing	34(4.8)	1(5.0)	
Professional titles (%)			
Resident	133(18.9)	7(35.0)	0.209
Attending physician	126(17.9)	4(20.0)	
Associate chief physician	222(31.5)	3(15.0)	
Chief physician	224(31.8)	6(30.0)	

### General information about pancreatic cancer

For the question of “what is the most common pathological type of pancreatic neoplasms”, only 361 surgeons (51.2%) selected “ductal pancreatic adenocarcinoma” and 285 (40.4%) selected the answer of “acinar cell carcinomas”. Although the differences were not significant, the hepato-biliary pancreatic surgeons had a 10% more correct answers (55%) than the surgeons majored in gastrointestinal surgery (45.8%) and general surgery (44.8%). There were no significant differences among surgeons at different ranks, from different levels of hospitals, or different regions.

The majority (81.4%) of surgeons thought that the incidence of PC in China was about 5–10%. Only 27.7% of the surgeons agreed that 80% of the PC patients were at the advanced stage when they were diagnosed. The resectability was considered <20% by 59.2% of the surgeons, 30–40% by 33.2% of the surgeons, and >50% by 5.8% of the surgeons.

### Other survey results

Tables 3, 4, 5, 6, 7 and 8 summarized the surgeons’ knowledge and opinions related to the environmental and genetic risk factors, clinical manifestations, TNM staging, diagnosis, resectability, and management of PC.

**Table 3:** Participants’ knowledge regarding environmental and genetic risk factors for PC

Risk Factors	Agree (%)	Disagree (%)	Unsure (%)	Missing (%)
Risk factors				
Family history	504(71.5)	74(10.5)	37(10.5)	90(12.8)
Cigarette smoking	522(74.0)	62(8.8)	27(3.8)	94(13.3)
Alcohol drinking	571(81.0)	50(7.1)	24(3.4)	60(8.5)
Type II diabetes	377(53.5)	137(19.4)	44(6.2)	147(20.9)
Chronic pancreatitis	594(84.3)	44(6.2)	19(2.7)	48(6.8)
Gallstone	349(49.5)	155(22.0)	58(8.2)	143(20.3)
Genetic susceptibility				
K-ras	542(76.9)	61(8.7)	15(2.1)	87(12.3)
P53	498(70.6)	84(11.9)	34(4.8)	89(12.6)
P16	305(43.3)	120(17.0)	63(8.9)	217(30.8)
BRCA	263(37.3)	163(23.1)	43(6.1)	236(33.5)

**Table 4:** Participants’ knowledge toward clinical manifestations of PC

Manifestations		Agree (%)	Disagree (%)	Unsure (%)	Missing (%)
Typical clinical manifestation	Stomachache, abdominal distension	582(82.6)	63(9.0)	11(1.6)	49(7.0)
	Abdominal mass	408(57.9)	146(20.7)	38(5.4)	113(16.0)
	Epigastrium tenderness	408(57.9)	146(20.7)	38(5.4)	113(16.0)
	Jaundice	586(83.1)	37(5.3)	24(3.4)	58(8.2)
	Gastrointestinal bleeding	166(23.6)	308(43.7)	54(7.7)	177(25.1)
Late manifestation	Flank and abdominal pain	635(90.1)	37(5.3)	14(2.0)	19(2.7)
	Fever	271(38.4)	238(33.8)	48(6.8)	148(21.0)
	Jaundice	588(83.4)	60(8.5)	14(2.0)	43(6.0)
	Ascites	528(74.9)	80(11.4)	22(3.1)	75(10.6)
	abdominal vascular murmur	176(25.0)	305(43.3)	103(14.6)	121(17.2)

**Table 5:** Surgeons’ knowledge about the staging of PC

Staging of Pancreatic Cancer		Agree (%)	Disagree (%)	Unsure (%)	Missing (%)
T-staging	T_X_: Unable to judge	553(78.4)	95(13.5)	7(1.0)	50(7.1)
	T_0_: No evidence of primary tumor	496(70.4)	168(23.8)	11(1.6)	30(4.3)
	T_1_: Tumor limited to the pancreas, ≤2 cm in longest dimension	567(80.4)	102(14.5)	9(1.3)	27(3.8)
	T_2_: Tumor limited to the pancreas, >2 cm in longest dimension	478(67.8)	163(23.1)	15(2.1)	49(7.0)
	T_3_: Extension beyond the pancreas, no involvement of the celiac or the superior mesenteric artery	534(75.7)	109(15.5)	27(3.8)	35(5.0)
	T_4_: Tumor involves the celiac or superior mesenteric artery	556(78.9)	106(15.0)	17(2.4)	26(3.7)
N-staging	N_X_: Unable to judge	540(76.6)	97(13.8)	7(1.0)	61(8.7)
	N_0_: No regional lymph node metastasis	612(86.8)	76(10.8)	5(0.7)	12(1.7)
	N_1_: Regional lymph node metastasis	623(88.4)	50(7.1)	5(0.7)	27(3.8)
M-staging	M_X_: Unable to judge	638(90.5)	22(3.1)	29(4.1)	16(2.3)
	M_0_: No distant metastasis	569(80.7)	45(6.4)	47(6.7)	44(6.2)
	M_1_: Distant metastasis	622(88.2)	58(8.2)	1(0.1)	24(3.4)

**Table 6:** Surgeons’ knowledge and opinion about the diagnosis of PC

Diagnosis of Pancreatic Cancer		Agree (%)	Disagree (%)	Unsure (%)	Missing (%)
Candidate tumor marker	CEA	412(58.4)	193(27.4)	15(2.1)	85(12.1)
	CA19–9	634(89.9)	50(7.1)	2(0.3)	19(2.7)
	CA242	313(44.4)	213(30.2)	18(2.6)	161(22.8)
Ultrasonograph (US)	Can be used to judge tumor size as the first line test	630(89.4)	57(8.1)	0(0.0)	18(2.6)
	has a high accuracy to detect PC less than 1 cm	171(24.3)	473(67.1)	6(0.9)	55(7.8)
	Low echoic mass is a sign of PC	560(79.4%)	100(14.2)	1(0.1)	44(6.2)
	Dilatation of the pancreatic duct is a sign of PC	463(65.7)	165(23.4)	4(0.6)	73(10.4)
	Dilatation of the common bile duct is a sign of PC	549(77.9)	98(13.9)	4(0.6)	54(7.7)
CT	Plain CT can be used to judge the location, size and boundary of the tumor	492(69.8)	163(23.1)	1(0.1)	49(7.0)
	Enhanced CT has a high accuracy to detect tumors <3 cm	604(85.7)	80(11.4)	3(0.4)	18(2.6)
	Can judge the extension of pancreatic cancer accurately	528(74.9)	109(15.5)	17(2.4)	51(7.2)
	Enhanced CT combined with 3-demension reconstruction of blood vessels is the best method to determine resectability	621(88.1)	46(6.5)	15(2.1)	23(3.3)
MRI	MRI is better than CT to detect and stage PC	328(46.5)	229(32.5)	1(0.1)	147(20.9)
	Good for detection of peripancreatic and lymphatic invasion	514(72.9)	133(18.9)	1(0.1)	57(8.1)
PET	A promising modality to differentiate malignant from benign lesions	483(68.5)	145(20.6)	2(0.3)	75(10.6)
	Can be used to judge the presence or absence of distant metastases	600(85.1)	78(11.1)	1(0.1)	26(3.7)
	High accuracy for resectability	365(51.8)	238(33.8)	2(0.3)	100(14.2)
pancreascopy	Best indicated for those could not be diagnosed by ERCP	520(73.8)	116(16.5)	3(0.4)	66(9.4)
	Good for early detection of PC	528(74.9)	131(18.6)	3(0.4)	43(6.1)
	Can be used to perform biopsy and cytology	605(85.8)	71(10.1)	5(0.7)	24(3.4)

**Table 7:** Surgeons’ knowledge and opinion towards the assessment of resectability of PC

	Agree (%)	Disagree (%)	Unsure (%)	Missing (%)
the resectability can be assessed by:				
CA19–9	392(55.6)	254(36.0)	6(0.9)	53(7.5)
CT	632(89.7)	56(7.9)	1(0.1)	16(2.3)
MRI	597(84.7)	77(10.9)	1(0.1)	30(4.3)
PET	445(63.1)	173(24.5)	9(1.3)	78(11.1)
ERCP	419(59.4)	208(29.5)	3(0.4)	75(10.6)
Selective angiography	506(71.8)	122(17.3)	4(0.6)	73(10.4)
CT Loyer stages^a^				
Type A: resectable	572 (81.1)	80(11.4)	18(2.6)	35(5.0)
Type B: resectable	534(75.7)	122(17.3)	5(0.7)	44(6.2)
Type C: resectable in half of patients	483(68.5)	146(20.7)	4(0.6)	72(10.2)
Type D: resectable in half of patients	438(62.1)	168(23.8)	8(1.1)	91(12.9)
Type E: non-resectable	411(58.3)	183(26.0)	4(0.6)	107(15.2)
Type F: non-resectable	540(76.6)	107(15.2)	8(1.1)	50(7.1)

Type A: Fat plane seperates the tumor and/or the normal pancreatic parenchyma from adjacent vessels; Type B: normal parenchyma separates the hypodense tumor from adjacent vessels; Type C: hypodense tumor is inseparable from adjacent vessels, and the points of contact form a concavity against the vessels; Type D: Hypodense tumor is inseparable from adjacent vessels, the points of contact form a concavity against the vessels or partially encircle the vessels. Type E: hypodense tumor completely encircles the vessel; Type F: hypodense tumor occludes the vessels

^a^reference [21]

**Table 8:** Surgeons’ knowledge and opinion toward the management of PC

Knowledge and opinion	Agree (%)	Disagree (%)	Unsure (%)	Missing (%)
Preoperative procedures				
No routine use of preoperative biliary drainage	632(89.7)	56(7.9)	1(0.1)	16(2.3)
The role of preoperative adjuvant therapy is to increase surgical resectability for R0 stage PC	536(76.0)	134(19.0)	2(0.3)	33(4.7)
Surgery for PC				
Laparoscopy might accurately detect peritoneal and hepatic dissemination	588(83.4)	89(12.6)	1(0.1)	27(3.8)
FNA can improve the diagnosis of PC	616(87.4)	60(8.5)	2(0.3)	27(3.8)
The scope of radical pancreatectomy				
(1) Bile duct beneath the middle of common hepatic duct and Peripheral lymph node	626(88.8)	59(8.4)	4(0.6)	16(2.3)
(2) The distal half of stomach, duodenum and 10 cm jejunum	576(81.7)	91(12.9)	4(0.6)	34(4.8)
(3) The soft tissues at the right side of superior mesenteric artery	557(79.0)	105(14.9)	2(0.3)	41(5.8)
(4) The soft tissues and peritoneum anterior to the inferior vena cava and partial aorta	513(72.8)	128(18.2)	3(0.4)	61(8.7)
Pancreatic stump management				
(1) Pancreaticojejunostomy is the canonical anastomosis.	638(90.5)	53(7.5)		14(2.0)
(2) If the pancreatic duct is dilated, pancreatic duct-to-mucosa anastomosis is feasible.	556(78.9)	92(13.1)	3(0.4)	54(7.7)
(3) If the stump of pancreas is soft with nondilated pancreatic duct, invaginated pancreaticojejunostomy is feasible.	587(83.3)	63(8.9)	4(0.6)	51(7.3)
Chemotherapy				
5-FU is the first-line chemotherapy	535(75.9)	126(17.9)	5(0.7)	39(5.5)
Gemcitabine is the first-line chemotherapy	589(83.6)	77(10.9)	6(0.9)	33(4.7)
Gemcitabine can improve the overall survival of advanced PC patients	546(77.5)	84(11.9)	6(0.9)	69(9.8)
Chemoradiation combined with chemotherapy will contribute to better outcomes than chemo- radiation therapy only	563(80.0)	75(10.6)	6(0.9)	61(8.7)

More than 70% of the participants agreed that family history, cigarette smoking, alcohol drinking, and chronic pancreatitis were the risk factors for PC. But only 50% of the participants thought diabetes was the risk factors. For the genetic mutations, surgeons understood better of K-ras and P53 than of p16 and BRCA. For the clinical manifestations, more than 80% of the participants agreed “stomachache and jaundice were typical clinical manifestation” for PC. Concerning genetic factors, nearly all the participants thought CA199 could be used as a marker for the diagnosis of PC. For the diagnosis of PC, although 67.1% surgeons did not agree “US had a high accuracy to diagnose the PC less than 1 cm”, 89.4% of the surgeons considered abdominal US as the first imaging modality to identify the tumor size. Most of the surgeons had a correct opinion of CT in diagnosis of PC. 85.7% of the surgeons agreed “enhanced CT had a high accuracy to detect tumors <3cm”, 74.9% of the surgeons agreed “CT can be used to judge the extension of PC accurately” and 88.1% of the surgeons approved “enhanced CT combined with 3-demension reconstruction of blood vessel is the best method to evaluate. Although 68.5% of surgeons thought PET can be used as a promising modality for noninvasive differentiation between benign and malignant lesions, 20.6% of the surgeons denied it. Half of the surgeons had the wrong idea that PET had a high accuracy of resectability. Surgeons’ knowledge about assessment of resectability was offered in Table 7. 89.7, 84.7 and 71.8% of the surgeons agreed that “CT, MRI and selective angiograph can be used to assess the resectability of PC”. But there were still 55.6, 63,1 and 59.4% of the surgeons had the wrong idea about CA199, PET and ERCP’s role in the assessment of resectability.

### Subgroup analysis

Surgeons’ responses to all the above questions did not depend on their geographic locations whereas surgeons working at level 3 hospitals had better knowledge in several areas. Compared to surgeons working at level 2 hospitals, higher percentage of surgeons working at level 3 hospitals considered smoking (86.8% vs 72.0%, *p* = 0.0002) and k-ras (88.9% vs 78.2%, *p* = 0.005) as risk factors of PC, agreed that ultrasound could be used to estimate tumor size as a screening tool (92.7% level 3 vs 85.6% level 2, *p* = 0.018), disagreed that “MRI is better than CT to detect and stage PC” (43.9% level 3 vs 22.7% level 2), agreed that “laparoscopy might accurately detect peritoneal and hepatic dissemination” (88.2% vs 78.0%, *P* = 0.007) and that “Gemcitabine is the first-line chemotherapy” (89.4% vs 80.9%, *P* = 0.011).

Surgeons’ response toward the following questions varies with professional ranks. A greater proportion of associate physicians (80.6%) than residents (71.4%) and chief physicians (67.9%) agreed that smoking was a risk factor for PC. The FDR adjusted *p* values were 0.036 and 0.026, respectively. However, more residents (82.0%) and attending physicians (81.0%) than associate (68.9%) and chief physicians (62.5%) considered family history a risk factor (*p* < 0.0001). Regarding resectability, fewer residents (78.7%) than others (89.6%) thought that “enhanced CT combined with 3-dimension reconstruction of blood vessels is the best method to evaluate the resectability of PC” (*p* = 0.003).

Finally, compared to surgeons specialized in pancreatic surgery, fewer other surgeons (82.2% vs 90.6%, *p* = 0.0005) had a correct answer regarding the definition of a T1 lesion. The same trend was observed regarding the pancreatic stump management and scope of radical pancreatectomy.

## Discussion

Pancreatic cancer is the eighth leading cause of cancer-related death [1]. Surgery resection may provide cure for early stages of cancer. Unfortunately, most pancreatic cancers are diagnosed late. The treatment of PC requires more than just technical skills. A full understanding of all aspects of PC oncology is necessary, including assessing risk factors, preoperative diagnosis and staging, surgical management, and post-operative adjuvant therapy. Studies have shown that there is a significant correlation between physician knowledge and decision-making [4, 5], as well as between surgeon knowledge and patient outcome [7]. These studies suggest that surgeons’ good overall knowledge about PC treatment contributes to the survival improvement of PC patients. However, surgeons’ understanding of the guidelines for the diagnosis and treatment of PC in China may vary. The present study demonstrated that most surgeons had a correct knowledge and opinion toward the risk factors, diagnosis and treatment of PC. But there were still some points, including (1) type 2 diabetes and genetic biomarkers of BRCA and their implication to PC diagnosis; (2) accuracy of US and CT in diagnosis of PC; (3) the markers for resectability assessment.

A recent study from China suggests that more than half of the pancreatic cancers may be preventable by adapting to a healthier life style [8], such as cessation of smoking and weight control. Our study showed that three quarters of the surgeons agreed smoking to be a risk factor and only half considered type 2 diabetes a risk factor. Diabetes mellitus has been implicated as both an early manifestation of pancreatic cancer and a predisposing factor [9]. Approximately 1% of diabetics aged more than 50 years will be diagnosed with pancreatic cancer within 3 years of first meeting criteria for diabetes [10]. Therefore, raising surgeons’ awareness about the association between type 2 diabetes and PC may increase the odds of early diagnosis.

Knowledge of genetic predisposition is of more than academic interest. Knowing the genetic markers associated with early development of PC may contribute to early detection. K-ras, p53 and p16 [11] may be associated with preneoplastic lesions and BRCA [12] has been considered as a screening test in high risk population. This study shows that surgeons had a good knowledge of K-ras and p53, but poor knowledge about p16 and BRCA. Improving their knowledge of genetic markers of PC seems necessary.

Pancreatic neoplasms can derive from different cell types and different types of neoplasms may have different cellular lineage and different clinicopathologic and biologic characteristics. The most common type of pancreatic neoplasms is ductal adenocarcinoma, contributing more than 90% of the carcinomas [13]. This study shows that only half of the surgeons had the correct answer. Forty percent of the surgeons thought acinar cell carcinomas were the most common type of pancreatic neoplasms.

Early and accurate diagnosis is the key to improve survival. Most PC patients present with nonspecific symptoms such as abdominal or back pain and jaundice depending on the tumor location [14]. The majority of surgeons (>80%) had the correct answer. It is surprising, however, more than half of the participants also thought that abdominal mass was a typical clinical symptom of PC.

When presented with these nonspecific symptoms, the appropriate next step is to perform an abdominal US as it is low-cost and noninvasive. Low echoic mass, dilatation of the pancreatic duct, and dilatation of the common bile duct are signs of pancreatic tumor [15, 16]. Most surgeons gave correct answers in this regard. Conventional ultrasound can only detect tumors >3 cm in diameter. A quarter of the participants, however, thought that US could detect tumors less than 1 cm. A significantly higher percentage of surgeons from level 3 hospitals than level 2 hospitals (92.7% vs 85.6%, *p* = 0.018) had the correct knowledge regarding US being the first line imaging study to evaluate tumor size. The reason may be that most of the pancreatic surgeons are graduated from top medical schools where they received high-quality medical education and clinical training.

CT is the most widely used imaging study to stage PC as well as to differentiate pancreatic adenocarcinoma from other pancreatic diseases. It is also useful to assess the resectability of PC [17]. More than 80% of surgeons correctly identified enhanced CT’s role in the diagnosis of PC, but nearly 70% of the surgeons incorrectly chose the answer of plain CT. Surgeons’ responses toward enhanced CT’s role in the assessment of resectability varied among different ranks. Fewer resident (78.7%) than other ranks (89.6%) correctly considered “enhanced CT combined with 3-dimension reconstruction of blood vessel is the best method to evaluate the resectability of PC” (*p* = 0.003). MRI is not superior to enhanced CT in the diagnosis of PC [18], but only one-third of surgeons answered this question correctly. A higher proportion of surgeons at level 3 hospitals (43.9%) than at level 2 hospitals (22.7%) gave the correct answer.

Enhanced CT has its limitations as well. It cannot differentiate PC less than 2 cm in diameter from chronic pancreatitis [19]. PET scan is a more accurate tool to differentiate malignant from benign lesions and can be used to detect distant metastasis [20]. The present study showed that one-fifth of surgeons were unaware of these advantages of PET scan. Surgeons’ knowledge regarding this relatively new technology needs improvement.

Staging of PC is one of the key issues in the management of PC patients. We use the same guidelines in China for staging, the American Joint Committee on Cancer/Union Internationale Contre Le Cancer Tumor-Node-Metastasis staging system (AJCC/UICC TNM). More than three quarters had correct answers for TNM staging except T0 (70.4%) and T2 staging (67.8%), suggesting that the participants had difficulties remembering the details.

The assessment of resectability is crucial. More than 80% of the surgeons correctly identified CT and MRI as the tools to assess resectability, but only 70% of them were aware of selective angiograph as an effective method. However, as selective angiograph is a traumatic opertation, it will be chosen second to the previous two. In addition, more than half of them incorrectly selected CA199, PET and ERCP. This knowledge gap needs to be improved.

Management of PC may include preoperative biliary drainage and adjuvant therapy in addition to surgery. More than 80% of the surgeons responded correctly regarding the management of PC. Higher proportion of surgeons working at high level hospitals had more correct answers than those at low level hospitals. Compared to surgeons specialized in pancreatic surgery, other groups had more incorrect answers regarding pancreatic stump management and scope of radical pancreatectomy. This may also because that most of the pancreatic surgeons are graduated from top medical schools where they received high-quality medical education and clinical training. So they have gained a more complete mastery of preoperative management of PC patients.

Our study is the first to evaluate the surgeons’ knowledge and opinion toward diagnosis and treatment of PC in China. Although this study had a good representation from all over China at different levels of hospitals and professional ranks, there are some limitations. For example, Surgeons who attended the 2011 China Surgical Week’s meeting in Beijing may have better knowledge regarding PC at the first place than those who did not attend. So the conlusion draw in our paper may narrow the gap between surgeons’ knowledge and the understanding of guidelines, which means an estensive survey across China will be essential. The questionnaire was designed based on the knowledge before 2011. As the recognition to PC developed during those years, the NCCN guideline for PC recommends MRI as an alternative option to CT for patients since 2013 which is opposite from our opinins in 2011. In addition, other limitations include controversies surrounding some of the questions that could have alternative answers, misinterpretation of questions, self-reported knowledge level and so on. All these limitations will lead to bias which need further revision of our questionnaire.

## Conclusions

In conclusion, most of the surgeons surveyed had good knowledge regarding the risk factors, diagnosis, and management of PC. However, there is considerable knowledge gap in certain areas and among different levels and ranks of surgeons. Programs designed for continuing medical education regarding pancreatic cancer to improve surgeons’ knowledge in China should be implemented.

## Additional file

Additional file 1: Questionnaire assessing surgeons’ knowledge about the diagnosis and treatment of PC. Questionnaire.doc Blank, English language version of the questionnaire used in data collection. (DOC 82 kb)

## Abbreviations

FDR: False discovery rate
PC: Pancreatic cancer
