# Supplementary material for: Surgeons’ knowledge regarding the diagnosis and management of pancreatic cancer in China: a cross-sectional study
Source: BMC Health Serv Res. 2017 Jun 9;17:395. doi: 10.1186/s12913-017-2345-6 (PMC5466735; doi:10.1186/s12913-017-2345-6)
Supplement: Additional file 1: — Questionnaire assessing surgeons’ knowledge about the diagnosis and treatment of PC. Questionnaire.doc Blank, English language version of the questionnaire used in data collection. (DOC 82 kb) [file 12913_2017_2345_MOESM1_ESM.doc]

01 Full name：____________________________

02 professional rank： ____________________________

03 specialty：______________________________

04 mobile phone：_________________________

04 practice hospital：_________________________

05 hospital’s level：Level 1 Level 2 Level 3

Choose the best answer for the following questions:

1. Agree 2. Disagree 3. Unsure

A01 The most common histology type for pancreatic cancer is:

(1)acinal adenocarcinoma

(2)ductal adenocarcinoma

(3)polymorphous adenocarcinoma

(4)fibrocyte adenocarcinoma

(5)mucinous carcinoma

A02 The incidence rate for pancreatic cancer in China is: (1)5/one hundred thousand

(2)10/one hundred thousand

(3)100/one hundred thousand

A03 How many peopole are in advanced stage when definitely diagnosed:

(1) 50% (2)60% (3)70% (4)80%

A04 At present, the rate of radical resection for pancreaic cancer is:

(1)below 15% (2)15%-20% (3)30%-40% (4)more than 50%

A05 which is the reason for its low radical resection rate:

(1) insidious onset

(2)rapid progression

(3)highly malignant

(4)low early diagnosis rate

A06 which are risk factors for pancreatic cancer：

(1)smoking

(2)drinking

(3)type 2 DM

(4)family history

(5)chronic pancreatitis

(6) Cholelithiasis

A07 which gene mutation is associated with pancreatic cancer?

(1)K-ras

(3)p53

(5)DPC4/Smad4

(2)p16

(4)BRCA

Choose the correct answer for the following questions:

1. Agree 2. Disagree 3. Unsure

B01 Which is the typical clinical manifestation for pancreatic cancer:

(1)Abdominal distention/pain

(3)Abdominal mass

(5)Jaundice

(2)upper abdominal tenderness

(4) digestive tract bleeding

B02 Which is the manifestation for pancreatic cancer in advanced stage:

(1)Abdominal/back pain

(3)Jaundice

(5)Fever

(2)Ascites

(4)Abdominal vessel murmur

B03 Which is correct for T staging of pancreatic cancer:

(1)Tx：Primary tumor cannot be assessed

(2)T0：No evidence of primary tumor

(3)T1：Tumor limited to the pancreas, 2 cm or less in greatest dimension

(4)T1：Tumor limited to the pancreas, 3 cm or less in greatest dimension

(5)T2：Tumor limited to the pancreas, more than 2 cm in greatest dimension

(6)T2：Tumor limited to the pancreas, more than 3 cm in greatest dimension

(7)T3：Tumor extends beyond the pancreas but without involvement of the aorta or the superior mesenteric artery

(8)T4：Tumor involves the aortaor the superior mesenteric artery

B04 Which is correct for N staging of pancreatic cancer:

(1)N x：cannot be assessed

(2)N 0：No regional lymph node metastasis

(3)N 1：Regional lymph node metastasis

B05 Which is correct for M staging of pancreatic cancer:

(1)Mx: Cannot be assessed (2)M0: No distant metastasis

(3)M1: Distant metastasis

Choose the correct answer for diagnosis of pancreatic cancer:

1. Agree 2. Disagree 3. Unsure

C01 Which cancer biomarker is associated with pancreatic cancer：

(1)CEA

(3)CAl9-9

(5)CA242

(3)AFP

(4)CA125

C02 Which is correct about ultrasound：

(1)Can be used to judge tumor size as the first line test

(2)has a high accuracy to detect PC less than 1 cm

(3)Low echoic mass is a sign of PC

(4)Dilatation of the pancreatic duct is a sign of PC

(5)Dilatation of the common bile duct is a sign of PC

C03 Which is correct about CT：

(1)Plain CT can be used to judge the location, size and boundary of the tumor

(2)Enhanced CT has a high accuracy to detect tumors <3cm

(3)Can judge the extension of pancreatic cancer accurately

(4)Enhanced CT combined with 3-demension reconstruction of blood vessels is the best method to determine resectability

C04 Which is correct about MRI:

(1)MRI is better than CT to detect and stage PC

(2)Good for detection of peripancreatic and lymphatic invasion

C05 Which is correct about PET-CT:

(1)A promising modality to differentiate malignant from benign lesions

(2)Can be used to judge the presence or absence of distant metastases

(3)High accuracy for resectability

C06 Which is correct about pancreascopy：

(1)Best indicated for those could not be diagnosed by ERCP

(2)Good for early detection of PC

(3)Can be used to perform biopsy and cytology

C07 Which can be used to assess the resectability：

(1)CA19-9

(2)CT

(3)MRI

(4)PET

(50ERCP

(6)Selective angiography

C08 Which is correct about Loyer stages：

(1)resectable：Fat plane seperates the tumor and/or the normal pancreatic parenchyma from adjacent vessels

(2)resectable: normal parenchyma separates the hypodense tumor from adjacent vessels

(3) borderline resectable: hypodense tumor is inseparable from adjacent vessels, and the points of contact form a concavity against the vessels

(4) borderline resectable: Hypodense tumor is inseparable from adjacent vessels, the points of contact form a concavity against the vessels or partially encircle the vessels

(5) unresectable：hypodense tumor completely encircles the vessel

(6) unresectable：hypodense tumor occludes the vessels

Choose the correct answer for preoperative treatment of pancreatic cancer:

1. Agree 2. Disagree 3. Unsure

D01 Which is correct about preoperative biliary drainage:

(1) No routine use of preoperative biliary drainage

(2) Biliary drainage when the blood bilirubin is more than 250mmol/L

(3) First-line procesure is ERCP

(4) First-line procesure is PTCD

D02 Which is correct about preoperative adjuvant therapy:

(1) adjuvant therapy is mianly for borderline resectable patients

(2) he role of preoperative adjuvant therapy is to increase surgical resectability for R0 stage PC

Choose the correct answer for surgical treatment of pancreatic cancer:

1. Agree 2. Disagree 3. Unsure

E01 Which is correct about laparoscopy:

(1) laparoscopy can explore the primary tumor

(2) Laparoscopy can find minimal metastasis to liver or peritoneal dissemination

(3) Laparoscopy can assess the vascular invasion

(4) Laparoscopy can avoid unnecessary open surgery

E02 Which is correct about FNA:

(1)intraopreative FNA can improve diagnostic rate

(2) intraopreative FNA can cause pancreatic fistula or bleeding

(3) intraopreative FNA should be made multidirection

(4)the positive rate for intraopreative FNA can reach 60%-80%

E03 Which is correct about the scope of radical pancreatectomy：

(1) Bile duct beneath the middle of common hepatic duct and Peripheral lymph node

(2) The distal half of stomach, duodenum and 10cm jejunum

(3) The soft tissues at the right side of superior mesenteric artery

(4) The soft tissues and peritoneum anterior to the inferior vena cava and partial aorta

E04 Which is correct about Pancreatic stump management:

(1) Pancreaticojejunostomy is the canonical anastomosis

(2) Pancreaticogastrostomy is the canonical anastomosis

(3) If the pancreatic duct is dilated, pancreatic duct-to-mucosa anastomosis is feasible

(4) If the stump of pancreas is soft with nondilated pancreatic duct, invaginated pancreaticojejunostomy is feasible

Choose the correct answer for of chemotherapy pancreatic cancer:

1. Agree 2. Disagree 3. Unsure

F01 Which is correct about first-line chemotherapy?

(1) 5-FU is the first-line chemotherapy

(2) Gemcitabine is the first-line chemotherapy

(3) Gemcitabine can not improve the overall survival of advanced PC patients

(4) Chemoradiation combined with chemotherapy will contribute to better outcomes than chemo- radiation therapy only
